# Polyarteritis nodosa with perirenal hematoma due to the rupture of a renal artery aneurysm

**DOI:** 10.1007/s13730-020-00552-z

**Published:** 2020-11-11

**Authors:** Taro Miyagawa, Yasunori Iwata, Megumi Oshima, Hisayuki Ogura, Koichi Sato, Shiori Nakagawa, Yuta Yamamura, Shinji Kitajima, Tadashi Toyama, Akinori Hara, Satoshi Kokubo, Norihiko Sakai, Miho Shimizu, Kengo Furuichi, Takashi Wada

**Affiliations:** 1 Department of Nephrology and Laboratory Medicine, Kanazawa University, 13-1 Takara-machi, Kanazawa, 920-8641 Japan; 2 Division of Nephrology, Kanazawa University Hospital, 13-1 Takara-machi, Kanazawa, 920-8641 Japan; 3 Division of Infection Control, Kanazawa University Hospital, 13-1 Takara-machi, Kanazawa, 920-8641 Japan; 4 Department of Medicine, Hakui General Hospital, 24 Matsusaki, Matoba-machi, Hakui, Ishikawa 925-8502 Japan; 5 Division of Blood Purification, Kanazawa University Hospital, 13-1 Takara-machi, Kanazawa, 920-8641 Japan; 6 Department of Nephrology, Kanazawa Medical University School of Medicine, 1-1 Daigaku, Uchinada, Kahoku, Ishikawa 920-0293 Japan

**Keywords:** Polyarteritis nodosa, Microaneurysm, Perirenal hematoma, Selective coil embolization

## Abstract

We present the case of a 67-year-old man in good health with perirenal hematoma due to a ruptured arterial aneurysm in the kidney. The patient developed weight loss, muscle weakness, multiple mononeuropathy, hypertension, anemia, renal insufficiency, and multiple lacuna infarctions about a month ago. He was admitted to the hospital due to worsening of his symptom. After admission, severe right-flank pain suddenly occurred; he was then transferred to our hospital. Renal angiography revealed bilateral multiple microaneurysms, and the patient was diagnosed with polyarteritis nodosa based on the clinical, radiographic, and histological findings. We performed selective coil embolization to the ruptured aneurysm and administered oral prednisolone along with intravenous methylprednisolone pulse therapy. Cyclophosphamide pulse therapy was also given. The treatment improved clinical and laboratory findings and achieved clinical remission. Selective coil embolization to the bleeding aneurysm of polyarteritis nodosa was minimally invasive and promptly effective. Immunosuppressants proved useful in the regulation of disease activity and the aneurysm.

## Introduction

Polyarteritis nodosa (PAN) is a systemic necrotizing vasculitis of small- or medium-sized arteries [1]. PAN is a rare form of vasculitis with a prevalence of approximately 31 cases per 1 million in Europe [2]. The mean age of onset is approximately 50 years. PAN is more common in men than in women [3]. Whereas most patients are idiopathic and negative for anti-neutrophil cytoplasmic antibodies (ANCA), some are associated with hepatitis B virus (HBV), hepatitis C virus (HCV), and human immunodeficiency virus (HIV) [4]. The diversity of clinical manifestations ranges from general symptoms, such as fever, weight loss, myalgias, and arthralgia, to organ-specific symptoms. The overall prognosis of PAN can improve with early diagnosis and administration of immunosuppressants, although untreated PAN still exhibits a poor prognosis.

The kidney, peripheral nervous system, and skin are frequently affected by PAN. Kidneys are involved in approximately 50% of cases. Representative manifestations include hematuria, proteinuria, recent-onset hypertension, and infarction, but PAN is not associated with glomerulonephritis [3, 5]. Whereas renal artery aneurysms are common, a perirenal hematoma is a rare complication in PAN [6].

In this report, we presented a case of PAN with unilateral perirenal hematoma due to a ruptured renal artery microaneurysm. Selective coil embolization to a unilateral perirenal hematoma by the ruptured microaneurysm was successfully conducted without complications. Our patient achieved clinical remission through treatment with oral prednisolone and intravenous methylprednisolone along with cyclophosphamide pulse therapy.

## Case report

A 67-year-old man in good health reported numbness and weakness of his lower legs in June 2010. He presented to the clinic due to the spread of the symptoms to his upper limbs. Magnetic resonance imaging revealed multiple lacuna infarctions. Laboratory analysis revealed anemia (hemoglobin [Hb], 9.8 g/dL) and renal dysfunction (creatine, 2.7 mg/dL). He was admitted to a nearby hospital for further examination and was diagnosed with hypertension and multiple mononeuropathy. In addition, laboratory findings revealed increased levels of C-reactive protein (CRP, 16.82 mg/dL). After 8 days in the hospital, severe right-flank pain suddenly occurred, and anemia worsened (Hb, 6.8 g/dL). Abdominal computed tomography (CT) without enhancement revealed a right perirenal hematoma. He was then transferred to our hospital for investigation and treatment.

**Fig. 1:**
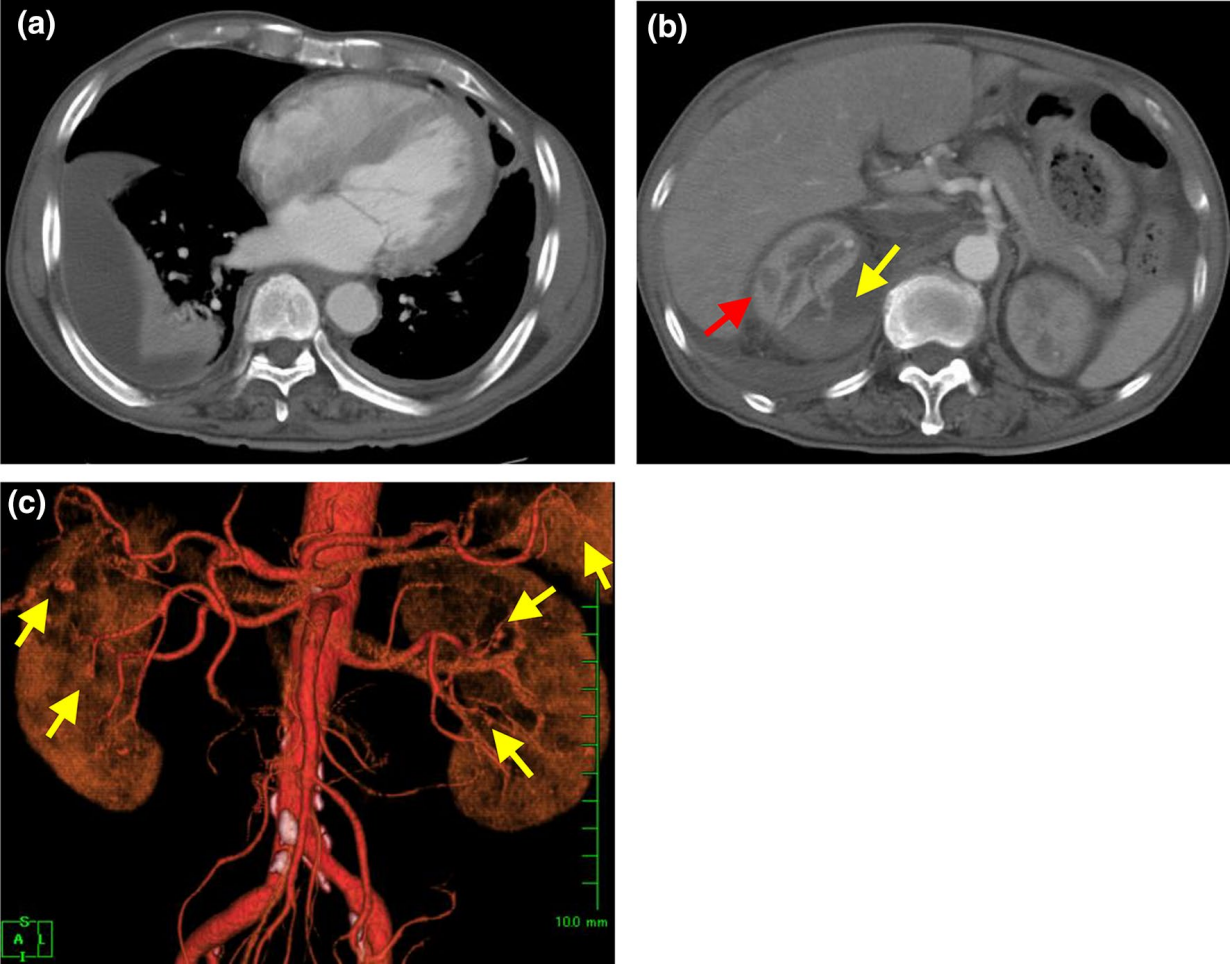
Contrast-enhanced computed tomography. **a** Bilateral pleural effusion was apparent. **b** CT scan image showing a right perirenal hematoma with extravasation of contrast media (yellow arrow) and a small renal infarction (red arrow). **c** CT angiography showing multiple small aneurysmal dilatations in the intrarenal branches of the bilateral renal arteries and in the intrasplenic branches of the splenic artery (yellow arrow)

**Fig. 2:**
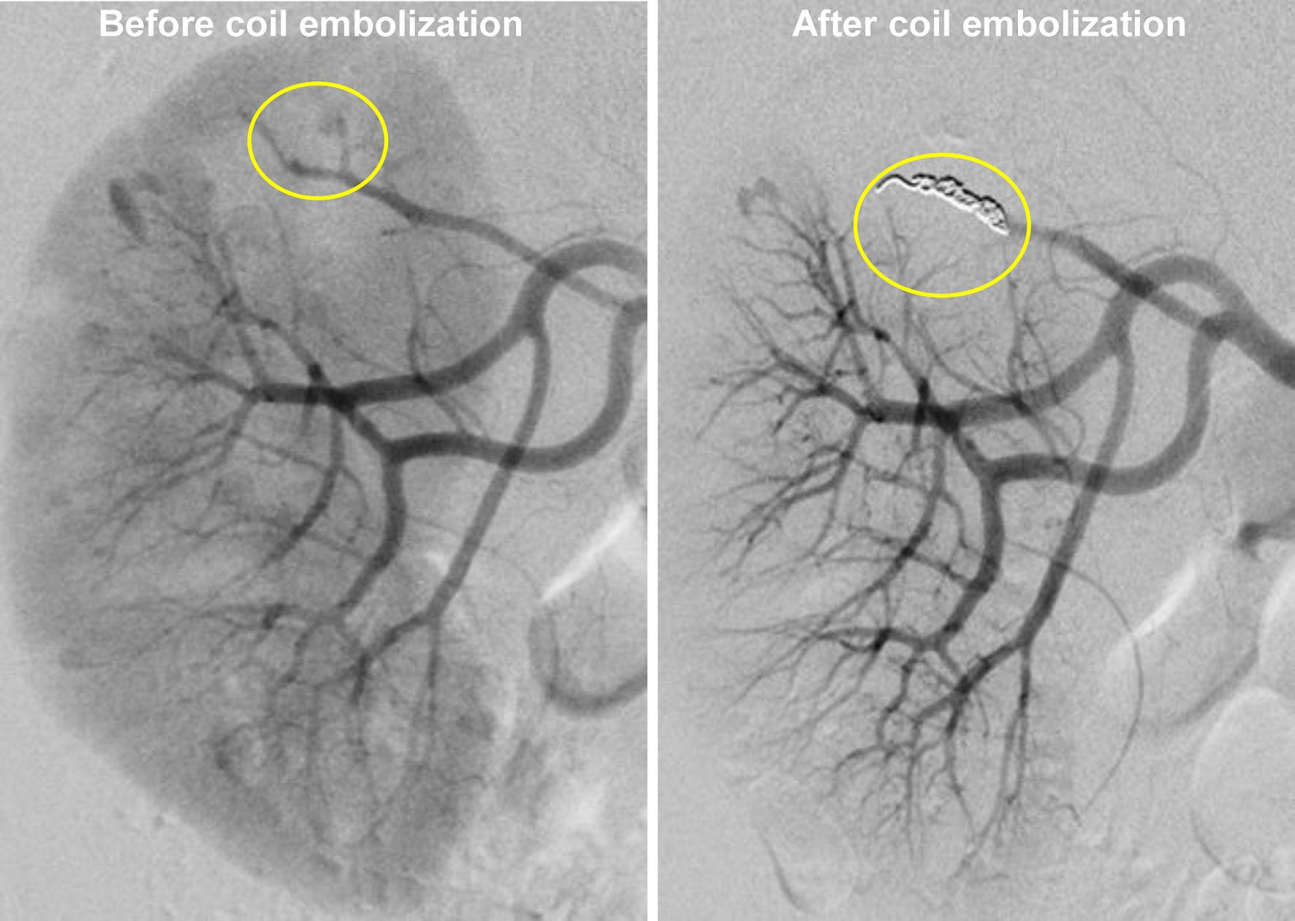
Selective coil embolization to the right renal artery aneurysm. Catheter angiography showing selective coil embolization to the bleeding renal artery aneurysm (yellow circle)

**Fig. 3:**
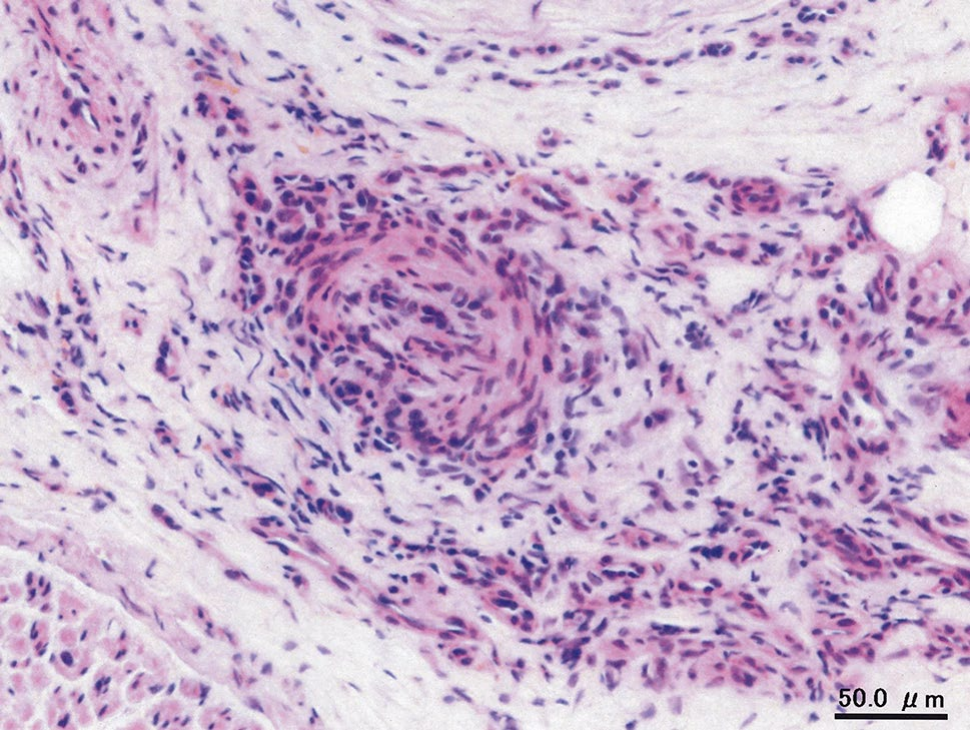
Left sural nerve biopsy. Mononuclear cell infiltration in small arteries with occlusion and recanalization of the vascular lumen (hematoxylin and eosin staining; original magnification × 400)

On admission, his height was 156.0 cm, and his weight was 54.5 kg (approximately 2-kg loss over months). His blood pressure was 192/114 mm Hg; pulse, 98 bpm; and body temperature, 37.1 ℃. On physical examination, palpebral conjunctiva was pallid, and he had severe right-flank pain with muscular defense. No skin rush was evident in the extremities, although hypesthesia was detected by neurologic examination. Laboratory findings on admission are presented in Table 1. The results of the urine tests were as follows: protein levels, 2.4 g/g⋅Cr; occult blood 3+ by dipstick test; red blood cells ≧ 100/high-power field; and sediment contained granular cast 2+ . The results of serum examinations were as follows: white blood count, 13,610/µL (with 74.0% neutrophils and 16.0% eosinophils); hemoglobin, 6.2 g/dL; platelet count, 32.1 × 10^4^/µL; alkaline phosphatase, 1,867 IU/L; γ-glutamyl transpeptidase (γ-GTP), 360 IU/L; blood urea nitrogen, 39 mg/dL; Cr, 1.3 mg/dL; erythrocyte sedimentation rate, 156 mm; C-reactive protein, 11.5 mg/dL; immunoglobulin (Ig) G, 2533 mg/dL; IgA, 306 mg/dL; IgM, 106 mg/dL; C3, 83 mg/dL; C4, 15 mg/dL; and CH50, 48 U/mL. ANA, PR3-ANCA, MPO-ANCA, anti-GBM antibody, and HBs-antigenemia were not detected. Contrast-enhanced CT revealed bilateral pleural effusion, right small renal infarction, and right perirenal hematoma. CT angiography revealed multiple small aneurysmal dilatations in the intrarenal branches of the bilateral renal arteries and the intrasplenic branches of the splenic artery (Fig. 1a–c). Emergency selective coil embolization was conducted to the right renal artery aneurysm, which was responsible for the bleeding (Fig. 2). To obtain a definitive diagnosis, we performed a left sural nerve biopsy (Fig. 3). We confirmed a small arterial occlusion with inflammatory cell infiltration in the sural nerve tissue, although a histological examination did not reveal fibrinoid necrosis or granuloma. Collectively, and based on his clinical and radiographic findings, we diagnosed polyarteritis nodosa.

**Table 1:** Laboratory data on admission

Urinalysis		Blood chemistry		Serology	
pH	5.5	TP	6.9 (g/dL)	ESR (1 h)	156 (mm)
Protein	2+	Alb	2.6 (g/dL)	CRP	11.5 (mg/dL)
	2.4 (g/g⋅Cr)	AST	32 (IU/L)	IgG	2533 (mg/dL)
Glucose	2+	ALT	14 (IU/L)	IgA	306 (mg/dL)
Occult blood	3+	ALP	1867 (IU/L)	IgM	106 (mg/dL)
RBC	≧100 (/HPF)	γ-GTP	360 (IU/L)	C3	83 (mg/dL)
WBC	10–19 (/HPF)	T-bil	0.6 (mg/dL)	C4	15 (mg/dL)
Hyaline cast	2+	UN	39 (mg/dL)	CH50	48 (U/mL)
Granular cast	2+	Cr	1.3 (mg/dL)	ANA	< 20
RBC cast	–	UA	7.9 (mg/dL)	PR3-ANCA	< 10 (EU)
Complete blood cell count		Na	133 (mEq/L)	MPO-ANCA	< 10 (EU)
WBC	13600 (/μL)	K	4.6 (mEq/L)	Anti-GBM antibody	< 10 (IU/mL)
Neu	74 (%)	Cl	103 (mEq/L)	RF	< 10 (IU/mL)
Lymph	7 (%)	Ca	7.8 (mg/dL)	Ferritin	680 (ng/mL)
Eos	16 (%)	IP	3.3 (mg/dL)	Cryoglobulin	–
Baso	0 (%)	T-Cho	123 (mg/dL)	HBs-Ag	–
Mono	3 (%)	TG	115 (mg/dL)	HBV-DNA	–
RBC	210×10^4^ (/μL))	HDL-Cho	23 (mg/dL)		
Hb	6.2 (g/dL)	BS	151 (mg/dL)		
Ht	18.6 (%)	LDH	209 (IU/L)		
Ret	9.2 (/μL)	Amy	43 (IU/L)		
Plt	32.1×10^4^ (/μL)	CK	25 (IU/L)		

*ESR* erythrocyte sedimentation rate, *ANA* anti-nuclear antibody, *PR3-ANCA* serine proteinase 3 anti-neutrophil cytoplasmic antibody, *MPO-ANCA* myeloperoxidase anti-neutrophil cytoplasmic antibody, *RF* rheumatoid factor, *GBM* glomerular basement membrane

**Fig. 4:**
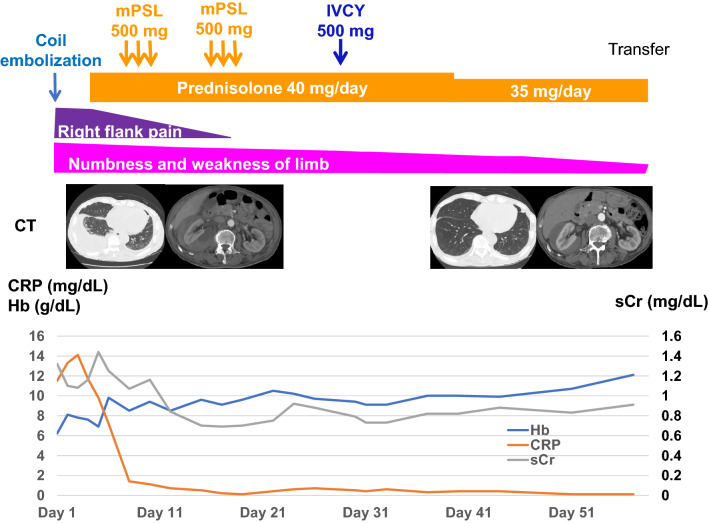
Clinical course of the case. *mPSL* methylprednisolone, *IVCY* intravenous cyclophosphamide

On the fifth hospital day, we started oral prednisolone therapy at 40 mg/day. On the 8th and 15th hospital days, intravenous methylprednisolone pulse (500 mg/day) therapy was started for 3 consecutive days. Although these therapies suppressed the disease activity, including inflammatory response, urine protein, and pleural effusion, the patient failed to achieve remission. Intravenous pulse cyclophosphamide therapy was, therefore, given on the 30th day. Clinical symptoms such as numbness and weakness of limbs gradually diminished, and laboratory findings dramatically improved. Moreover, contrast-enhanced CT revealed the disappearance of pleural effusion and the decreased size of perirenal hematoma concomitant with aneurysms. The patient was then transferred to another hospital for rehabilitation training (Fig. 4).

## Discussion

Here, we presented a case of PAN with a unilateral perirenal hematoma due to a ruptured arterial aneurysm.

PAN is diagnosed based on clinical manifestations, angiography, and histopathology. Our patient was healthy but had developed weight loss, muscle weakness, multiple mononeuropathies, hypertension, anemia, renal insufficiency, and multiple lacuna infarctions for about a month. He suffered from a sudden onset of right-flank pain with a perirenal hematoma due to a ruptured renal aneurysm. He also exhibited bilateral pleural effusion without an elevated ANCA titer. The presence of three or more American College of Rheumatology (ACR) criteria revealed a sensitivity of 82% and specificity of 86% in the cases with PAN [7]. Our patient fulfilled 5 of the 10 ACR criteria and could have also been diagnosed with PAN based on the Chapel Hill criteria [1]. Consistent with this case, 93% of ANCA-negative patients with arteriographic anomalies were diagnosed as PAN rather than microscopic polyangiitis in a review of 949 patients with systemic vasculitis [8].

Our patient presented with a unilateral perirenal hematoma due to a ruptured renal artery aneurysm. In a survey of 348 patients with PAN, 104 (66.4%) had kidney microaneurysms [5]. In addition, the frequency of aneurysms in PAN increased with clinical severity [9]. Hypertension has been reported as a risk of aneurysm rupture [10]. In this case, hypertension had not been revealed in an annual medical checkup, indicating that it occurred in a short period. Renal hemorrhages are typically caused by an arterial aneurysmal rupture and rarely by renal artery dissection or rupture [6]. Spontaneous perirenal hematoma was first reported as a complication of PAN by Schmidt in 1908 [11]. Although a perirenal hematoma caused by an aneurysmal rupture is a common complication, the frequency of renal artery dissection or rupture-induced perirenal hematoma is relatively low [6]. Mortality is reportedly 50%, and the recurrent bleeding rate is 18% with nephrectomy therapy [6]. Currently, transcatheter selective renal artery embolization is typically performed instead of nephrectomy. With this therapy, favorable outcomes have been reported, regardless of embolic material, unilateral, or bilateral hematoma [6, 12–15]. The procedure-related mortality of selective embolization is 3.6% [16]. The frequencies of micro-renal infarction, lateral abdominal pain, and fever are 17.6%, 11.8%, and 5.9%, respectively [17]. Fortunately, in our patient, selective coil embolization to the ruptured aneurysm was conducted successfully with no complications.

In 1996, the prognostic Five-Factor Score (FFS) was established to evaluate the outcomes and mortality of PAN, Churg–Strauss syndrome [18]. The revised FFS (2011) indicates the prognosis of PAN by four factors: age (older than 65 years), renal insufficiency (serum creatinine > 1.7 mg/dL), cardiac insufficiency, and severe gastrointestinal involvement [19]. The FFS of the case was 2, based on multiple bilateral renal aneurysms and failure to achieve remission by glucocorticoid monotherapy. A previous report indicated that cyclophosphamide therapy may improve microaneurysms of PAN [20] and is recommended for cases of PAN with an FFS ≧ 1 as induction therapy [3]. We, therefore, administered cyclophosphamide, resulting in remission. The 5-year survival of PAN has improved from 13% in untreated patients to approximately 80% in patients treated with glucocorticoid and cyclophosphamide [5, 21, 22]. However, as the 5-year survival rate in PAN with an FFS ≧ 2 is 65.0% [5], careful follow-up is necessary.

In summary, a case of PAN with unilateral perirenal hematoma due to a ruptured renal artery microaneurysm was described. Selective coil embolization to the ruptured renal microaneurysm was minimally invasive and promptly effective. In addition, therapy with immunosuppressants resulted in clinical remission.
